# Standardizing postpartum family planning counseling guidance in Ghana: A stepped-wedge cluster randomized implementation effectiveness trial

**DOI:** 10.1371/journal.pone.0340482

**Published:** 2026-01-30

**Authors:** Sarita Sonalkar, Ernest Maya, Chris Guure, Arden McAllister, Dzifa Puplampu, Roseline Doe, Robert Gallop, James Kiarie, Mary Eluned Gaffield

**Affiliations:** 1 University of Pennsylvania Perelman School of Medicine, Philadelphia, Pennsylvania, United States of America; 2 Department of Population, Family, and Reproductive Health, School of Public Health, University of Ghana, Legon-Accra, Ghana; 3 Department of Biostatistics, School of Public Health, University of Ghana, Legon-Accra, Ghana; 4 Department of Global Health and Population, Harvard T.H. Chan School of Public Health, Boston, Massachusetts, United States of America; 5 Reproductive, Maternal, Newborn, Child, and Adolescent Health (RMNCAH), Healthy Ageing, UHC-Life Course Cluster, World Health Organization, Roman-Ridge- Accra, Ghana; 6 World Health Organization, Geneva, Switzerland; The University of Texas at San Antonio School of Public Health, UNITED STATES OF AMERICA

## Abstract

**Background:**

Postpartum family planning can reduce morbidity and mortality for parents and children, however, up to 62% of birthing people have an unmet need for contraception due to implementation challenges. In this study, we aimed to evaluate implementation and effectiveness of the Postpartum Family Planning Package, a multifaceted implementation strategy combining provider use of the World Health Organization Medical Eligibility Criteria Mobile App (WHO MEC app), provider education, and counseling restructuring on the postnatal ward, to promote individualized family planning counseling prior to hospital discharge after childbirth.

**Methods:**

We conducted a stepped-wedge trial in the Greater Accra and Eastern regions of Ghana. The Postpartum Family Planning Package implementation strategy was introduced sequentially at three public hospitals. We used a generalized linear mixed effects model to adjust for the time variable via the random effects part of the model, controlling for all other independent variables. Additionally, we assessed for time by intervention interaction.

**Results:**

From 5^th^ October 2020–1^st^ October 2021, we enrolled 2096 patients and 191 providers. Post-intervention encounters were more likely to include discussion of all appropriate postpartum family planning methods compared to pre-intervention encounters (63% vs 39%). Patients counseled individually post-intervention were four times more likely to have all appropriate family planning methods discussed (aOR 4.28; 95% CI 2.35, 7.78). A family planning method decision was made before discharge in 49.5% of post-intervention encounters, compared to 18.3% pre-intervention (aOR 4.45; 95% CI 2.85, 6.93). Individual counseling was associated with higher uptake of family planning methods prior to discharge (aOR 1.74; 95% CI 1.04, 2.91).

**Conclusion:**

Implementation of the Postpartum Family Planning Package resulted in high fidelity to the intervention and was effective in promoting patient decision to select contraceptive methods postpartum. Future research should examine the effect of our strategy when used during antenatal and all other postpartum encounters, as well as mechanisms to improve method uptake.

## Introduction

Family planning has a critical impact on maternal and child morbidity when it results in couples spacing their pregnancies more than two years apart [1,2]. Modern contraceptives are safe and effective, and international guidelines exist for their delivery in the postpartum period [3]. However, unmet need for postpartum family planning persists [4], exposing an implementation gap.

An essential component to decreasing short interpregnancy interval is ensuring counseling and access to family planning methods prior to discharge from a facility after childbirth [5]. However, the postpartum period is a complex time for family planning counseling. Recommendations on the timing, safety, and need for hormonal and non-hormonal contraceptive initiation are influenced by dynamic factors including venous thromboembolism risk, breastfeeding status, and competing priorities such as emphasis on lactational amenorrhea and dual HIV/pregnancy prevention. For patients in Africa, hesitation to use modern contraceptives may stem from concerns about side effects and health risks, inconvenience, and belief that contraception is unnecessary while breastfeeding [6]. A 2016 Cochrane review of educational interventions in the postpartum period concluded that although some interventions appeared to influence contraceptive uptake and pregnancy prevention, interventions could be strengthened through improved program design and implementation [7].

Outside the postpartum period, implementation strategies involving education and infrastructure support have been shown to improve family planning uptake [8], and mobile health interventions have been found to be a feasible adjunct in improving health outcomes [9]. Job aids, particularly mobile job aids, are an increasingly important part of family planning service delivery [10,11]. The World Health Organization (WHO) Postpartum Family Planning Compendium is a mobile application that aids providers to make evidence-based postpartum family planning recommendations, and was pilot tested in Accra, Ghana [3,12]. The results from our work on this mobile application informed the development of the WHO Medical Eligibility Criteria for Contraceptive Use Mobile Application (WHO MEC app; (S1 Fig), which has a dedicated section on postpartum contraception.

Prior research in Ghana showed that postpartum family planning method uptake was higher when family planning counseling was done at the postnatal ward prior to discharge after childbirth compared to outpatient counseling (58% vs 52%) [13]. The 2022 WHO Postnatal Care Guidelines recommend that healthy birth parents who deliver in a health facility and their newborns should receive postnatal care within the facility for at least 24 hours [14], which should allow enough time for family planning counseling. Given the widespread use of the WHO MEC in country programs [15], we sought to study an implementation strategy that incorporates a mobile adaptation of the WHO MEC.

In this research, we aimed to evaluate implementation and effectiveness of the Postpartum Family Planning Package (PFPP), a multifaceted implementation strategy combining the WHO MEC app, provider education, and counseling restructuring on the postnatal ward, to promote individualized family planning counseling prior to hospital discharge after childbirth. Our primary implementation outcome was fidelity, defined as documented discussion by a postnatal provider of all guideline-appropriate contraceptive methods. The primary effectiveness outcome was family planning method received by the patient prior to discharge. We used a stepped-wedge cluster randomized controlled design to allow initiation of the implementation strategy in phases, improving the feasibility of implementation, and to allow all sites to have the benefit of the intervention [16]. The Consolidated Framework for Implementation Research (CFIR) guided our study design, analysis, and interpretation [17].

## Methods

This study was conducted at three “clusters” or district hospitals in Ghana: Maamobi General Hospital, Ga West Municipal Hospital, and Nsawam Government Hospital. These hospitals were selected due to existing relationships between investigators and their leadership. Prior to beginning the study, investigators also met with leadership to ensure that the study operations would be supported. In addition, sites were chosen based on geographic location and the presence of active family planning and labor and delivery services. All providers and patients involved in the study provided written informed consent. Institutional permission for recruitment and observation was obtained from each site. This research was also approved by three institutions: World Health Organization Ethics Review Committee (certified protocol number A65990), the Ghana Health Service Ethics Review Committee (GHS-ERC: 15/08/19), and the University of Pennsylvania Institutional Review Board (protocol 834804). We registered this trial on ClinicalTrials.gov (NCT04306029). Study activities took place between 5^th^ October 2020–1^st^ October 2021.

### Baseline postpartum family planning counseling practices

On the postpartum wards, family planning counseling was provided primarily in a group setting for postpartum patients who were to be discharged that day. Standard care at the start of the study was for patients desiring family planning methods in the immediate postpartum period after group counseling to be referred to family planning clinics after discharge. Each hospital had an outpatient family planning clinic staffed by midwives and nurses co-located on its premises. All methods, including implants, intrauterine devices (IUDs), progestin-only injectables, and oral contraceptive pills, could be provided at the family planning clinic, and sterilization was provided in the hospital prior to discharge. All methods remained in stock throughout the study period.

### Study design

**Fig 1:**
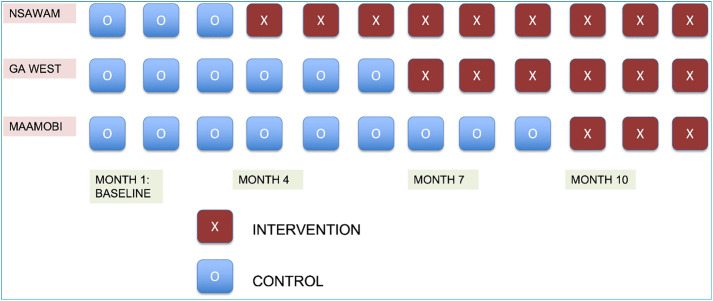
Schematic of stepped-wedge study design.

We conducted a prospective hybrid type 2 implementation and effectiveness study using a stepped-wedge cluster randomized controlled trial design [18] to evaluate implementation outcomes and clinical effectiveness of the PFPP. The intervention was counseling using the WHO MEC app, which includes nearly all informational content of the WHO MEC, and detailed information about each family planning method. The WHO MEC app contains a provider-facing tool to guide patients based on preferences for method attributes (such as privacy or associated bleeding patterns). In addition, the WHO MEC app informs providers as to whether method eligibility for potentially preferred methods is satisfied based on the MEC. We developed an implementation strategy including education, staff engagement, and restructuring of counseling to a one-on-one model that was initiated sequentially, every three months, at each hospital in random order for one year [18]. Random allocation to timing of rollout was conducted by an investigator and biostatistician not involved in data collection (RG). Baseline data collection at all facilities started three months prior to initiating the intervention. Data collection occurred at all sites throughout the roll-out of the intervention (Fig 1). The cluster was defined as the hospital, and a total of four steps were included with the first as the baseline. After each 3-month step, one additional random hospital received the intervention, without allocation concealment.

### Study participants

The primary study participants were service providers (mainly midwives and nurses) and excluded students. The secondary participants were newly postpartum patients who agreed for their interaction with providers to be observed. We excluded very ill postpartum patients. Service providers were continuous participants throughout the study period, while the postpartum patients were participants only in distinct time periods (pre-implementation or post-implementation).

### Training of research coordinators

Study staff at the University of Ghana School of Public Health trained three research coordinators/interviewers during a pretesting phase. The research coordinators had completed masters-level education, had more than 5 years work experience, and prior experience in qualitative and quantitative data collection. The training included objectives of the study, ethics involving human research, Ghana Health Service COVID-19 prevention protocols, data collection forms, and staff observation and feedback. Each coordinator was assigned to one facility where they observed inpatient postpartum encounters by service providers. We also recruited one family planning midwife per site who worked at the facility’s family planning unit as the clinical lead to serve as the link between their facility and the study team.

### Data collection approach

Unblinded research coordinators observed clinical postpartum care using a standardized checklist and counseling daily during one randomly selected week of each month at each included hospital. A block randomization scheme was used such that the week of the month that each site was observed was balanced among the sites over the study period. Blinding of the observer was not possible as the clinical encounters included use of the WHO MEC app after the training had taken place.

We collected baseline information on providers, including provider role, gender, and number of years in practice. Coordinators collected data on elements of provider counseling in real time while observing the provider-patient encounter. Included in the data collection forms were objective measures of whether all appropriate contraceptive methods were discussed, which individual methods were discussed, whether patients were given instructions on how to access the method, and recommendations for birth spacing (S1 File). From the medical record, coordinators documented patient method choice, as well as any medical comorbidities or birth complications that might have affected method choice (S2 File). We held biweekly team meetings to understand challenges and share experiences, and data was regularly monitored to ensure consistency.

Coordinators called all enrolled postpartum study patients approximately six weeks after discharge from the hospital to assess uptake after discharge. They also reviewed the clinical records to log family planning methods distributed to study patients.

### Implementation strategy: The Postpartum Family Planning Package (PFPP)

The PFPP included two components: education regarding postpartum family planning counseling, and restructuring of counseling to a one-on-one, individualized approach.

### Education

We conducted a full-day in-person training session for postpartum nurses and midwives on best practices in family planning counseling. We included educational content derived from the WHO MEC for Contraceptive Use, and each individual provider received coaching in downloading the WHO MEC app. We educated providers on concepts from the Balanced Counseling Strategy Plus Toolkit [19], patient-centered shared decision-making techniques [20], postpartum contraceptive methods, safety of contraceptive methods in the postpartum period [21], and used interactive small group case-based education to practice the content learned using the WHO MEC app. The training sessions were facilitated by the investigators, one Ghanaian consultant Obstetrician-Gynecologist (OB/GYN) and three Ghanaian specialist OB/GYNs who had subspecialist training in family planning. The training materials were developed by the investigators with input from the trainers. Clinical leads, including nurse managers and family planning nurse midwives, provided regular on-site coaching, content refreshers, and support [22].

### Restructuring of postpartum family planning counseling to a one-on-one model

As a part of the PFPP, we asked postpartum providers to counsel patients one-on-one at the bedside using the WHO MEC app rather than in a group setting. Nurse managers were engaged throughout the research period and conveyed expectations to staff to use the WHO MEC app during counseling and to provide counseling individually. Family planning midwife trainers, who worked at the family planning clinics located on the hospital campus, provided periodic supportive supervision to nurses on the ward during the course of the study. One-on-one counseling lasted from 5–15 minutes, and while there was potential for this to take additional time compared to the prior model, our qualitative research revealed that use of the WHO MEC App allowed these sessions to be efficient and streamlined [22].

### Sample size determination

For power and sample size calculations, we assumed recruitment of 50% of eligible patients, with a baseline of 10% of patients already receiving counseling regarding all guideline-appropriate methods during the hospitalization for delivery prior to the initiation of the implementation strategy [23,24]. We assumed a type I error of 5%, three steps (one for each time a new site was introduced to the PFPP), and four time points. Using these assumptions, we expected to observe at least 1716 encounters over one year, which would allow us 80% power to detect a 12.5% or greater increase in the percent of patients who receive appropriate counseling [25]. If the percent of patients receiving counseling regarding all guideline-appropriate methods prior to implementation of the package is 20^% [^23^]^, we would have 80% power to detect a 16.5% or greater increase in percent of patients who received appropriate counselling.

### Statistical analysis

We computed descriptive statistics for each of the included variables. A generalized linear mixed effects models (GLMM) approach was used to adjust for the time variable via the random effects part of the model while all other independent variables, including the cluster (facility as a nuisance parameter), were controlled for via the fixed effects aspect of the model. GLMM yielded estimated odds ratios with 95% confidence intervals. We retained a variable in the model if it influenced the estimate of the primary exposure variable, the outcome, or both. We specified a type I error level of 20% for variable inclusion in the GLMM, while the statistical significance of the exposure variables of interest was set at the typical type I error level of 5% significance testing. Additionally, we assessed for time by intervention interaction, as each hospital was introduced into the intervention arm each quarter of the year. All statistical analyses were carried out via complete case approach because the assumption of Missing Completely at Random (MCAR) was appropriate. An assessment of multicollinearity was carried out using variables’ tolerance and variance inflation factors (VIF). All the VIF values were below 2, an indication that multicollinearity was not a significant concern. All analyses were performed using the STATA programming language (Stata 14 – StataCorp LP).

### Predictors

We included individual-level predictor variables based on their significance at the bivariate level analyses, and their clinical significance. These variables were as follows: site of enrollment, mode of delivery, gestational age at delivery (preterm or full term), patient age, marital status, educational status, and number of live children. Educational status was included as a proxy for socioeconomic status.

### Outcomes

#### Implementation outcomes.

All outcomes were measured via direct observation of counseling encounters by research coordinators and documented during the counseling discussion. The primary implementation outcome was fidelity, defined as the documented discussion of all seven appropriate postpartum family planning methods (progestin-only pill, implant, injectable, IUD, lactational amenorrhea, barrier methods, and sterilization). In addition, we documented fidelity of counseling practices, including when to start the method, how to use the method, side effects, health or pregnancy risks, effectiveness, and recommendations for birth spacing, all of which were prompted by the WHO MEC app. We also measured reach, defined as the proportion of encounters in which the WHO MEC app was used for counseling, and the proportion of encounters in which one-on-one counseling was performed.

#### Effectiveness outcomes.

The primary effectiveness outcome was whether a chosen family planning method was received by the patient before leaving the facility. In addition, we measured family planning method chosen prior to discharge (regardless of receipt), and use of a method by 6 weeks postpartum.

#### Patient and public involvement.

The design of this study emerged from pilot research engaging local clinicians, academia, and the Ghana Health Service in which we iteratively developed a mobile tool for postpartum family planning counseling. Patients were not directly involved in the design of the study.

#### Inclusivity in global research.

Additional information regarding the ethical, cultural, and scientific considerations specific to inclusivity in global research is included in the Supporting Information (S3 File).

## Results

**Fig 2:**
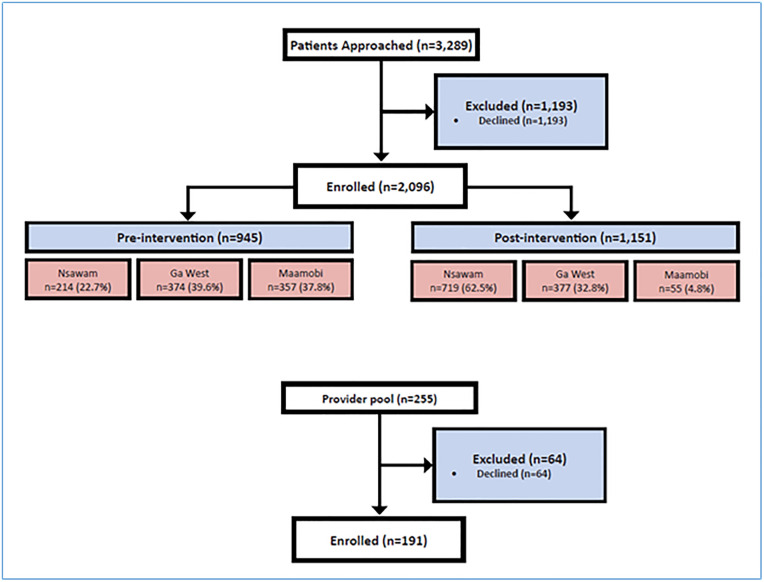
Study flow.

We enrolled provider and patient participants between 5^th^ October 2020 and 1^st^ October, 2021. Of 255 providers, 191 participated. Of 3289 patients approached, 2096 were enrolled, 945 pre-intervention and 1151 post-intervention (Fig 2). Reason for declining participation was not collected. Baseline characteristics at the three hospitals differed by proportion of cesarean deliveries, provider type, and provider years in practice (Table 1).

**Table 1:** Baseline hospital characteristics and provider characteristics.

	Nsawam	Ga West	Maamobi
**Hospital characteristics**			
Total deliveries (n)	6817	4111	2229
Cesarean deliveries (n, %)	2525 (37.0)	1388 (33.8)	664 (29.8)
Preterm deliveries (n, %)	354 (5.2)	59 (1.4)	56 (2.5)
**Provider characteristics (n)**			
Healthcare providers enrolled	47	75	69
Provider type			
Physician	0	0	0
Midwife	46	64	63
Nurse	1	10	3
Other^a^	0	1	3
**Number of years in practice (n)**			
0-4	35	33	21
5-9	8	21	14
10 or more	4	21	34

^a^Other providers included one community health nurse, one ward assistant, and two students (inadvertently included).

For the post-intervention time periods, Nsawam, Maamobi and Ga West recruited 62.5%, 32.8% and 4.8% of patient participants respectively, representing 55% of the total (pre- and post-implementation) enrolled patients. Differences in proportions of patients enrolled reflected differences in caseloads across sites. Most patients had spontaneous vaginal delivery, had at least primary education, and delivered at full term. Post-intervention patients were on average younger than pre-intervention patients (mean age 28.9 years compared with 29.5 years, respectively). All patients were biologically assigned female at birth; we did not collect data on gender identity. In general, irrespective of the intervention phase, patients were healthy and had few medical comorbidities (Table 2).

**Table 2:** Baseline patient characteristics.

	Pre-intervention (n = 945)	Post-intervention (n = 1151)
**Patients observed, n (%)**		
Nsawam	214 (22.6)	719 (62.5)
Ga West	374 (39.6)	377 (32.7)
Maamobi	357 (37.8)	55 (4.8)
**Vaginal mode of delivery, n (%)**	646 (68.4)	665 (57.8)
**Gestational age, n (%)**		
Preterm <36 weeks	29 (3.1)	59 (5.1)
Not Preterm >=36 weeks	916 (96.9)	1092 (94.9)
**Age of birthing parent, mean (SD)**	29.5 (6.1)	28.9 (6.2)
**Marital status, n (%)**	854 (90.3)	947 (82.3)
**Educational status, n (%)**		
No education	50 (5.3)	32 (2.8)
Primary	118 (12.5)	164 (14.2)
Junior high	406 (43.0)	558 (48.5)
Senior high	266 (28.1)	283 (24.6)
Tertiary	105 (11.1)	114 (9.9)
**Gravidity, median (IQR)**	3 (2,4)	3 (2,4)
**Parity, median (IQR)**	2 (1,4)	2 (1,4)
**Induced abortions, median (IQR)**	1 (1,2)	1 (1,2)
**Spontaneous abortions, median (IQR)**	1 (1,1)	1 (1,2)
**Living children, median (IQR)**	2 (1,4)	2 (1,4)
**Any medical comorbidity, n (%)**	115 (12.2)	124 (10.8)
**Gestational age at delivery,** m**edian (IQR)**	39 (38,40)	39 (38,40)
**Breastfeeding initiation prior to discharge, n (%)**	904 (95.7)	1093 (95.0)
**Baby discharged with parent, n (%)**	878 (92.9)	1054 (91.6)

SD, standard deviation.

IQR, interquartile range.

### Bivariate analysis

#### Implementation outcomes.

Our primary implementation outcome was documented discussion of all seven appropriate postpartum family planning methods available in Ghana (progestin-only pill, implant, injectable, IUD, lactational amenorrhea, barrier methods, and sterilization) during observation of counseling by research coordinators. The WHO MEC app was used in all post-intervention encounters. One-on-one counseling was done in 8% of pre-intervention encounters and 77% of post-intervention encounters. Sixty-three percent of post-intervention patients engaged in discussion of all appropriate postpartum family planning methods with their provider compared to 38.7% of pre-intervention patients (OR 1.53; 95% CI 0.88, 2.64; Table 3).

**Table 3:** Fidelity of postpartum family planning counseling pre-and-post-intervention and associated unadjusted and adjusted odds ratios.

	Pre-Intervention n = 945	Post-Intervention n = 1151	Unadjusted OR	Adjusted OR
	n (%)	n (%)	[95% CI]	[95% CI]
**All appropriate family planning methods discussed**	366 (38.73)	724 (62.90)	1.53 [0.88, 2.64]	0.16 [0.08, 0.31] ^^,^ ***
**Interaction effect: intervention period by counseling type (one-on-one vs group)**	n/a	n/a	n/a	4.28 [2.35, 7.78]^^,***
**Discussion of when to start method**	917 (97.04)	1127 (97.91)	0.46 [0.10, 2.00]	0.71 [0.12, 4.23]
**Discussion of how to use method** ^**#**^	213 (22.54)	684 (59.43)	6.91 [0.33, 14.49]	1.02 [0.03, 33.74]
**Discussion of side effects**	338 (35.77)	790 (68.64)	15.78 [11.14, 22.36] ***	6.51 [4.10, 10.34] ***
**Discussion of risks**	156 (16.51)	294 (25.54)	161.34 [65.6, 396.83] ***	86.08 [12.39, 598.18] ***
**Discussion of effectiveness**	928 (98.20)	1140 (99.04)	1.90 [0.88, 4.07]	1.21 [0.10, 14.84]
**Discussion of recommendations for birth spacing discussed**	939 (99.37)	1148 (99.74)	2.45 [0.61, 9.80]	1.94 [0.50, 7.62]

* p < 0.5; ** p < 0.01; *** p < 0.001

Table 3 legend: Odds ratios were adjusted for site of enrollment, mode of delivery, gestational age at delivery (preterm or full term), patient age, marital status, educational status, and number of living children. This estimate represents the effect of the intervention when counseling was conducted in a group setting, the reference category for the interaction term. The interaction effect shows that the combination of the intervention period and one-on-one counseling led to a 4.28-fold increase in the odds of discussing all appropriate methods. # Outcome values were missing for one study site; results reflect findings from Nsawam and Ga West Hospitals only.

#### Effectiveness outcomes.

Our primary effectiveness outcome was proportion of patients who received a family planning method prior to discharge. Prior to the intervention, 18% of patients expressed a choice of a family planning method at the time of discharge, and 5.0% received a method prior to discharge. After the intervention, 50% of patients in the post-intervention group expressed a choice of method prior to discharge, and 7.5% of patients received a method (Table 5). Pre-intervention, the most requested methods were injectables (4.6%), implants (4.0%), and sterilization (6.0%). Post-intervention, the most requested methods were implants (14.4%), injectables (9.7%), and sterilization (8.6%), and the most common methods received were sterilization and implants.

**Table 4:** Association of one-on-one (vs. group) counseling with implementation outcomes, adjusting for covariates and study site.

	Unadjusted OR [95% CI]	Adjusted OR [95% CI]
**All appropriate family planning methods discussed**	5.38 [3.79-7.64] ***	0.53 [0.26-1.08]
**Discussion of when to start method**	0.17 [0.08-0.36] ***	0.17 [0.17-0.38] **
**Discussion of how to use method** ^**#**^	18.23 [10.52-31.61] ***	20.43 [11.50-36.30] ***
**Discussion of side effects**	4.41 [3.54-5.50] ***	1.28 [0.77-2.13]
**Discussion of risks**	4.67 [3.47-6.27] ***	1.61 [0.84-3.08]
**Discussion of effectiveness**	1.30 [0.61-2.80]	0.50 [0.07-3.63]
**Discussion of recommendations for birth spacing discussed**	1.40 [0.21-9.42]	0.72 [0.13-4.01]

Table 4 legend: The reference is group counseling. Odds ratios were adjusted for site of enrollment, mode of delivery, gestational age at delivery (preterm or full term), patient age, marital status, educational status, and number of living children. For the Unadjusted Analysis, one-on-one counseling was compared to group counseling across the entire study period, pooling pre- and post-intervention data. The Adjusted Analysis includes the following covariates: site, mode of delivery, gestational age, patient age, marital status, educational status, and number of living children, and time trend. # Outcome values were missing for one study site; results reflect findings from Nsawam and Ga West Hospitals only. * p < 0.5; ** p < 0.01; *** p < 0.001

**Table 5:** Association of intervention on effectiveness outcomes and one-on-one counseling type on effectiveness outcomes.

	Pre-Intervention N = 945	Post-Intervention n = 1151	Unadjusted OR	Adjusted OR [95% CI]
	n (%)	n (%)	[95% CI]	[95% CI]
**Association of intervention on effectiveness outcomes**				
Any method chosen prior to discharge	173 (18.31)	570 (49.52)	4.62 [3.14, 6.80] ***	4.45 [2.85, 6.93] ***
Any method received before discharge	47 (5.00)	86 (7.50)	1.00 [0.67, 1.48]	0.80 [0.45, 1.41]
Any method received by 6 weeks	60 (6.35)	112 (9.7)	1.17 [0.77, 1.76]	1.11 [0.62, 1.95]
**Association of one-on-one counseling type on effectiveness outcomes**				
Any method chosen	–	–	1.56 [1.34, 1.78] ***	2.31 [1.76, 3.03] ***
Any method received before discharge	–	–	1.45 [0.88, 2.38]	1.74 [1.04, 2.91] *
Any method received by 6 weeks	–	–	1.32 [0.91, 1.91]	1.33 [0.84, 2.90]

* p < 0.5.

*** p < 0.001.

Table 5 legend**:** The reference for odds ratios is group counseling. Odds ratios were adjusted for site of enrollment, mode of delivery, gestational age at delivery (preterm or full term), patient age, marital status, educational status, and number of living children. Data were missing for 44.1% of participants at 6 weeks.

### Multivariable analysis

#### Implementation outcomes.

In the multivariable analysis, we included the following variables as confounders: site of enrollment, mode of delivery, gestational age at delivery (preterm or full term), patient age, marital status, and number of living children. Given the multi-component nature of the implementation strategy, we assessed for interaction of pre- and post-intervention status and group versus one-on-one counseling on the primary outcome of fidelity. Post-intervention providers were significantly more likely to undertake one-on-one rather than group counseling, as this was a key component of the intervention (OR, 30.17; 95% CI 20.05, 45.39). Thus, we employed the use of an interaction effect to assess the effect of both variables on fidelity. The main effect for the intervention period (aOR 0.16; 95% CI 0.08, 0.31) represents the effect of the intervention when counseling was delivered in a group setting, and as such this was not considered to be representative of the effect of the full package. When accounting for the receipt of one-on-one counseling using an interaction effect, patients in the post-intervention group who also received one-on-one counseling were four times more likely to receive counseling on all appropriate family planning methods (OR, 4.28; 95% CI 2.35, 7.78) compared with patients who received counseling pre-intervention (Table 3). Other elements of fidelity included discussion of when to start the method, how to use the method, side effects, health or pregnancy risks, effectiveness, and recommendations for birth spacing. Patients in the post-intervention group were significantly more likely to receive counseling on side effects (aOR, 6.51; 95% CI 4.10, 10.34) and risks (aOR 86.08; 95% CI 12.39, 598.18) of contraceptive methods (Table 3).

Patients who received one-on-one counseling were more likely to have discussions of how to use methods (aOR 20.43; 95% CI 11.50, 36.30) compared with those in group settings but were less likely to receive counseling on when to start the method (aOR 0.17; 95% CI 0.17, 0.38; Table 4).

For many estimates, we observed a large difference between unadjusted and adjusted odds ratios. These were primarily due to adjustment for the confounding effect of study phase (pre- vs. post-intervention), which was strongly associated with both the likelihood of receiving one-on-one counseling and the primary outcome. The adjusted model isolates the effect of counseling type independent of the overall intervention period.

#### Effectiveness outcomes.

Adjusted for confounders, patients in the post-intervention group were four times more likely to choose a family planning method prior to discharge (OR, 4.45; 95% CI 2.85, 6.93) compared to the pre-intervention group, but they were no more likely to receive a method before discharge or to be using a method at six weeks postpartum compared to the pre-intervention group (Table 5). The adjusted odds of choosing a method prior to discharge after undergoing one-on-one counseling were twice as high compared with group counseling (OR 2.31; 95% CI 1.76, 3.03; Table 5). In the adjusted model, there was a 74% increase in the odds of patients receiving a method before discharge if one-on-one counselling occurred (OR, 1.74; 95% CI 1.04, 2.91; Table 5). Statistical interaction effects between the intervention group and one-on-one counseling were not significant in the analysis of effectiveness outcomes. At 6 weeks postpartum, 455 participants (22%) reported using a method, but most (384; 84%) were using the lactational amenorrhea method.

## Discussion

### Principal findings

In this stepped-wedge randomized trial, we found that the PFPP – a multifaceted implementation strategy involving provider education and individualized counseling using the WHO MEC app – significantly improved provider counseling on guideline-appropriate family planning methods in the immediate postpartum period. In addition, this intervention resulted in a significantly increased proportion of patients making a choice about a family planning method prior to discharge from the hospital, with implants and injectables most likely to be chosen. While actual method uptake prior to discharge in the post-intervention group was not statistically significant, we did note an association between one-on-one counseling and receipt of a method, signaling the importance of individualized family planning support. When controlling for the effect of the intervention period and other covariates, one-on-one counseling alone was not associated with a statistically significant increase in the discussion of all methods. This suggests that the *primary driver* of the improvement in fidelity was the entire package of the intervention (training, use of the WHO MEC app, and emphasis on one-on-one counseling), not just the switch to a one-on-one format by itself.

### Context

Our intervention uniquely included the WHO MEC app alongside an education and individualized counseling intervention. In low- and middle-income countries, providers have demonstrated interest in integrating apps into the primary care setting [26,27], but their effect on clinical outcomes has not previously been demonstrated. Our research group’s contextual inquiry and pilot data showed acceptability of the use of a mobile application and allowed us to improve the contraceptive app to meet the needs of health care providers [12]. A 2018 study of patients in the Central region of Ghana showed that only 18.5% of patients were using postpartum family planning in the first month after delivery, and the mean time of first contraceptive uptake following the last birth among patients who used contraceptives was 3.5 months [23]. Data from Nepal, Sri Lanka, and Tanzania provide support that postpartum family planning interventions directed toward providers can improve both counseling and uptake of family planning methods [24,28,29]. However, a secondary analysis of data from a cluster randomized trial of a postpartum IUD intervention in Tanzania showed that while the intervention improved counseling and uptake of IUDs, counseling on any non-IUD method was reduced. This finding supports the need for interventions like the PFPP that are focused on the full method mix to ensure autonomy in family planning choice. The PFPP is scalable for providers given the publicly availability of the mobile application. Indeed, we conducted a qualitative study of providers who participated in this research, which showed that overall, the various elements of the intervention, especially the WHO MEC app, were perceived as feasible, acceptable, and appropriate [22].

Group counseling has been shown to have benefits in prenatal care, and the prenatal period may be an appropriate time for group family planning counseling given that the purpose might be primarily for information sharing [30,31]. However, our findings suggest that individual counseling may be more appropriate for the postnatal time period given that a private counseling discussion tailored to personal preferences and medical conditions will allow for choice and receipt of a method prior to discharge. Furthermore, it is possible that family planning counseling is better suited to an individualized model regardless of timing, given the preference-sensitive nature of the discussion needed.

### Clinical implications

The results of this study support providers using a package of the WHO MEC app along with education and individualized counseling to increase family planning knowledge and decision-making in the immediate postpartum period.

### Research implications

Future research should explore the gap between patient family planning method choice and delivery to assess the factors that contributed to overall low uptake of methods prior to discharge. This may include contraceptive supply, health system factors such as clinic staffing or appointment availability, or patient-level factors such as stigma, fear, perception that lactational amenorrhea is most appropriate immediately after delivery, transportation or other logistical concerns, among many others. Furthermore, our research shows that implants and injectables are most highly desired in our research population, both of which can be placed without significant restructuring of space or personnel in a postpartum ward setting. Qualitative studies of patients in the postpartum period may elucidate some of the reasons for low uptake. Finally, the incremental added value of the WHO MEC app to interventions of provider education and individualized counseling was not elucidated in the current study.

### Policy and future directions

Our research has important policy implications for the dissemination of family planning guidance. As digital tools become more accessible and their use becomes more prevalent in low resource settings, it is evident that digital guidance is essential for evidence dissemination. With front-line providers having access to the WHO MEC app, they essentially had the entire WHO MEC guideline at their fingertips. The vast WHO MEC app information offered ‘refresher’ information to support healthcare providers to counsel patients with confidence. Our study findings show that provider education enhanced with digital tools improves delivery of evidence-based information. While the WHO MEC app is widely available and free of cost, scalability of its use in clinical care as well as scalability of one-on-one counseling and our educational intervention should be further studied. Updated WHO Postnatal Care Guideline recommendations recommend up to four health care provider contacts until six weeks postpartum and recommend provision of comprehensive contraceptive information and services during postnatal care [14]. Given that the WHO MEC app significantly increased comprehensiveness of counseling and selection of a contraceptive method in the immediate postpartum period, policy makers should build upon these findings and encourage the use of the WHO MEC app at all postnatal care encounters.

Future directions include studying the PFPP in lower resource and rural settings and expanding the package beyond the hospital discharge period to include subsequent postnatal visits. Our findings illustrate the imperative for strengthened postpartum family planning counseling and provision as an essential component of the additional postnatal care contacts now recommended by the WHO.

### Strengths and limitations

Our study’s strengths include its stepped-wedge methodology employing a large sample size and randomization of the order of intervention rollout and of observed weeks in the ward. Engagement from health system leadership as well as healthcare providers allowed us to meet recruitment goals, and for staff to adhere to implementation strategies with a high level of fidelity. Real-time observation of contraceptive counseling allowed us insight into practices that would not always be captured through medical record documentation. Finally, our preparation for this research through team building across three countries and piloting and revision of the mobile app contributed to the engagement of the international team.

A significant limitation of our research is that both providers and patients may have altered their behaviors in response to observation by research coordinators. Given our primary implementation outcome of fidelity, or discussion of all appropriate family planning methods with the patient, this was an essential aspect of our study design. However, we realize that providers may not have completed a full discussion of methods if research staff were not present. In addition, we did not include technical training on point-of-care placement of IUDs or implants. While these methods remained available throughout the study period, implementation strategies for their placement on the ward were not included in our intervention. Because data were collected based on observation of provider counseling, our ability to understand how patients understood the counseling was limited. A further limitation relates to the 36% of approached women who declined participation. Unfortunately, the study did not collect specific reasons for their refusal, and so we acknowledge that this decline rate indicates that our enrolled sample may represent a subset of postpartum women who were more comfortable with being observed, which could limit the generalizability of our findings.

## Conclusions

Our study shows that implementation of the PFPP resulted in high fidelity to the intervention and effectiveness in promoting patient contraceptive decision-making in the postpartum period. Future research should investigate the reasons behind the gap between patient method selection and actual uptake before discharge. There is also a need to examine the effect of our strategy at lower-level health facilities, during antenatal and all postpartum encounters, and to develop mechanisms to improve method uptake.

## Supporting information

S1 Fig Screenshot of the WHO MEC app. (TIF)

S1 File Postpartum counseling observation checklist. (PDF)

S2 File Client chart extraction tool. (PDF)

S3 File Inclusivity-in-global-research-questionnaire. (DOCX)

S4 File Postpartum family planning dataset. (XLS)
