# Supplementary figures and images for: Standardizing postpartum family planning counseling guidance in Ghana: A stepped-wedge cluster randomized implementation effectiveness trial
Source: PLoS One. 2026 Jan 30;21(1):e0340482. doi: 10.1371/journal.pone.0340482 (PMC12857993; doi:10.1371/journal.pone.0340482)

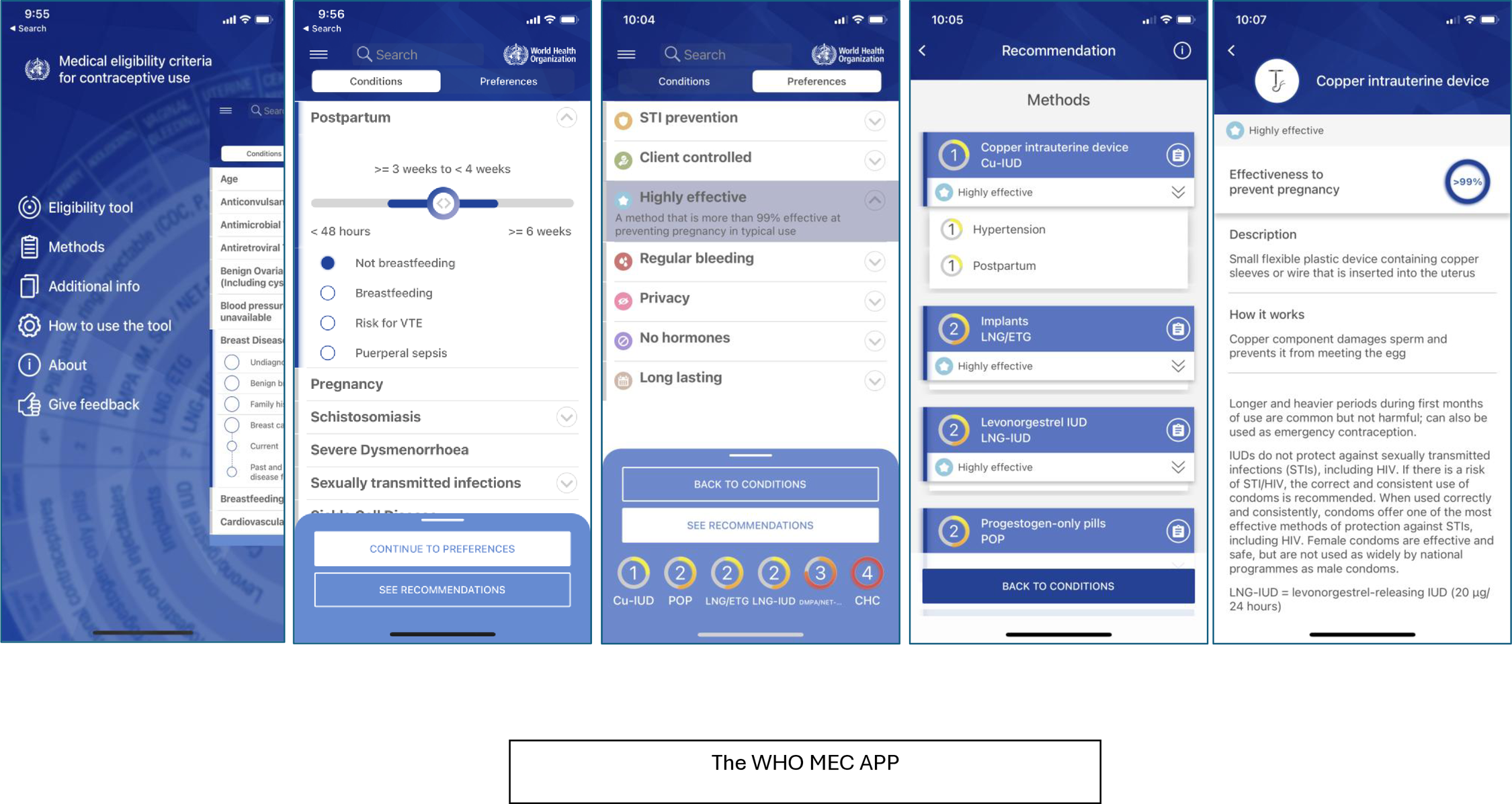

Supplement: S1 Fig — (TIF) [file pone.0340482.s001.tif]
