# Supplementary material for: Standardizing postpartum family planning counseling guidance in Ghana: A stepped-wedge cluster randomized implementation effectiveness trial
Source: PLoS One. 2026 Jan 30;21(1):e0340482. doi: 10.1371/journal.pone.0340482 (PMC12857993; doi:10.1371/journal.pone.0340482)
Supplement: S1 File — (PDF) [file pone.0340482.s002.pdf]

Postpartum Counseling Observation

Encounter Information

Client Subject ID: [record_id]
Client Name: [client_first_name] [client_last_name]
MRN: [client_mrn]
DOB: [client_dob]

Postpartum Encounter Date

____

Provider Subject Number

____

Observational Data Collection

Was this counseling done individually or with a group of clients?

- ◯ Individually
- ◯ With a group of clients

Were medical conditions of the client reviewed by provider?

- ◯ Yes
- ◯ No

Were recommendations for birth spacing discussed?

- ◯ Yes
- ◯ No

Were family planning methods discussed?

- ◯ Yes
- ◯ No

If yes, which family planning methods were discussed?

- ☐ Condoms
- ☐ Sterilization
- ☐ Copper IUD / Loop
- ☐ LNG IUD / Mirena
- ☐ Implant
- ☐ DMPA (injection) - intramuscular (the type you get in the clinic)
- ☐ DMPA (injection) - subcutaneous (the type you can give to yourself)
- ☐ Combined Oral Contraceptive (COC) Pills
- ☐ Progesterone Only Pill (Minipill)
- ☐ LAM (using exclusive breastfeeding to prevent ovulation)
- ☐ Other

(Check all that apply)

If other, please explain

____

Was a family planning method chosen by client at the time of consult?

- ◯ Yes
- ◯ No

If yes, what type of family planning method was chosen?	☐ Condoms<br>☐ Sterilization<br>☐ Copper IUD / Loop<br>☐ LNG IUD / Mirena<br>☐ Implant<br>☐ DMPA (injection) - intramuscular (the type you get in the clinic)<br>☐ DMPA (injection) - subcutaneous (the type you can give to yourself)<br>☐ Combined Oral Contraceptive (COC) Pills<br>☐ Progesterone Only Pill (Minipill)<br>☐ LAM (using exclusive breastfeeding to prevent ovulation)<br>☐ Other<br>(Check all that apply)
If other, please explain what type of family planning method was chosen:	__________________________
If NO, was there a reason why method was not chosen?	◯ Does not desire contraception due to desiring pregnancy<br>◯ Does not desire contraception for other reasons<br>◯ Undecided on method<br>◯ Unknown<br>◯ Other
Other reason why method was not chosen:	__________________________
Was the counseling app used?	◯ Yes<br>◯ No
If the counseling app was used, how was it used?	☐ Provider reference<br>☐ Patient counseling<br>☐ Teaching trainees or other providers<br>☐ Other<br>(Check all that apply)
Other:	__________________________
Was client given clear instructions on how to access the method?	◯ Yes<br>◯ No
If yes, what kind of instructions was the client given?	◯ Told to follow up with family planning<br>◯ Sterilization performed<br>◯ IUD placed<br>◯ Implant placed<br>◯ DMPA given<br>◯ Prescription given for pills<br>◯ Other
If other, please explain	__________________________

Which of the following method-based counselling was done by provider? Select all that may apply:	☐ When to start method<br>☐ How to use<br>☐ Side effects<br>☐ Risks<br>☐ Effectiveness<br>☐ Other<br>☐ None - no method-based counselling was done<br>(Check all that apply)
If other, please explain	__________________________
Progress Notes:	________________________________
