# Supplementary material for: Standardizing postpartum family planning counseling guidance in Ghana: A stepped-wedge cluster randomized implementation effectiveness trial
Source: PLoS One. 2026 Jan 30;21(1):e0340482. doi: 10.1371/journal.pone.0340482 (PMC12857993; doi:10.1371/journal.pone.0340482)
Supplement: S2 File — (PDF) [file pone.0340482.s003.pdf]

Client Chart Extraction

Client Record ID ______________________

Client Subject ID: [client_subject_number]
Client Name: [client_first_name] [client_last_name]
MRN: [client_mrn]
DOB: [client_dob]

Final Study Status
- ◯ Delivered during week of observation
- ◯ Withdrew from study
- ◯ No observation completed

Reason for withdrawal ______________________

Initials of study staff completing chart review: ______________________

Pregnancy Record (p.4)

Gravidity (total number of pregnancies) ______________________

Parity (total number of children delivered) ______________________

Number of induced abortions ______________________

Number of spontaneous abortions ______________________

Number of preterm births ______________________

Number of stillbirths ______________________

Number of live children ______________________

Medical and Surgical History (p.5)

Client's medical conditions (select all that may apply):

- [ ] Hypertension
- [ ] Heart disease
- [ ] Sickle cell disease
- [ ] Diabetes
- [ ] Epilepsy
- [ ] HIV infection
- [ ] Asthma
- [ ] Respiratory disease
- [ ] Tuberculosis
- [ ] Mental illness
- [ ] Smoking (recorded in "Social Risk Factors" section)
- [ ] Other
- [ ] NONE RECORDED

(Check all that apply)

If other, please explain

____

Delivery Record (beginning p.21)

Weeks of pregnancy (gestational age--weeks)

____
(Enter 999 if unknown)

Date of Delivery

____

Type of delivery:

- ◯ Normal
- ◯ Vacuum
- ◯ Cesarean section
- ◯ Other

Describe other type of delivery:

____

Indication for vacuum / cesarean

____

Were there any labor and delivery complications recorded?

- ◯ Yes
- ◯ No

Please describe complications

____

Delivery Outcome

- ◯ Live Birth
- ◯ Stillbirth
- ◯ Early Neonatal Death

Discharge Summary (beginning p.22)

Breastfeeding/breast milk initiation?
◯ Yes
◯ No

Baby suckling established
◯ Yes
◯ No

Baby's condition at maternal discharge
◯ Normal
◯ Abnormal

If Abnormal, please describe:
____

Postnatal Records for Mother

Did the client have a contraceptive method chosen in their Maternal and Child record book before discharge?
◯ Yes
◯ No

Which contraceptive method was chosen?
☐ Condoms
☐ Sterilization
☐ Copper IUD / Loop
☐ LNG IUD / Mirena
☐ Implant
☐ DMPA (injection) - intramuscular (the type you get in the clinic)
☐ DMPA (injection) - subcutaneous (the type you can give to yourself)
☐ Combined Oral Contraceptive (COC) Pills
☐ Progesterone Only Pill (Minipill)
☐ LAM (using exclusive breastfeeding to prevent ovulation)
☐ Other
(Check all that apply)

If other, please explain
____

Did client access the method by 6 weeks/42 days postpartum? (obtained from either Maternal and Child book or Family Planning records)
◯ Yes
◯ No

Which method did the client receive?
◯ Condoms
◯ Sterilization
◯ Copper IUD / Loop LNG IUD / Mirena Implant
◯ Implant (Implanon/ Jadelle)
◯ DMPA (injection) - intramuscular (the type you get in the clinic)
◯ DMPA (injection) - subcutaneous (the type you can give to yourself)
◯ Combined Oral Contraceptive (COC) Pills
◯ Progesterone Only Pill (Minipill)
◯ LAM (using exclusive breastfeeding to prevent ovulation)
◯ Other (Check all that apply)

Other:
____

Number of days postpartum when client accessed method:	____________________ (Enter 0 if day of delivery)
Where did the client obtain the method?	◯ In the hospital<br>◯ at the family planning clinic<br>◯ at the pharmacy<br>◯ other
If other, please explain	____________________
When did the client obtain the method?	◯ Before hospital discharge<br>◯ After hospital discharge
If before hospital discharge, where:	◯ On the postpartum ward<br>◯ At the family planning clinic<br>◯ At the pharmacy<br>◯ Other
If other, please explain	____________________
If after hospital discharge, where:	◯ At the family planning clinic<br>◯ At the pharmacy<br>◯ Other
If other, please eplain	____________________
Progress Notes:	_________________________
